# Discovery of conserved peptide-MHC epitopes for directly alloreactive CD8^+^ T cells

**DOI:** 10.3389/frtra.2025.1525003

**Published:** 2025-01-29

**Authors:** Alexandra E. Hill, Eric T. Son, Moumita Paul-Heng, Chuanmin Wang, Shivanjali Ratnaseelan, Martina Denkova, Pouya Faridi, Asolina Braun, Anthony W. Purcell, Nicole A. Mifsud, Alexandra F. Sharland

**Affiliations:** ^1^ Transplantation Immunobiology Group, Sydney Medical School, University of Sydney Faculty of Medicine and Health, Sydney, NSW, Australia; ^2^ Department of Biochemistry and Immunity Program, Monash Biomedicine Discovery Institute, Monash University, Melbourne, VIC, Australia

**Keywords:** allorecognition, peptide-MHC, CD8^+^ T cell, epitope discovery, heart transplant, acute rejection, tetramer screening

## Abstract

Mass Spectrometry allied with *in-vivo* generation of activated alloreactive T cell populations and tetramer screening facilitates the identification of endogenous peptides that are directly recognised in complex with allogeneic Major Histocompatibility class I (MHC I) molecules by alloreactive CD8^+^ T cells. We had previously used this approach for the discovery of immunogenic self-peptides presented by the allomorph H-2K^b^ (K^b^). In this study, we identified 22 highly immunogenic self-peptides presented by H-2K^d^ (K^d^). Peptide abundance across skin, spleen and liver samples (estimated as the product of the spectral intensity obtained for these samples) was the principal factor influencing recognition of peptide-K^d^ epitopes. Predicted binding affinity (BA score) and overall peptide hydrophobicity were also independently correlated with immunogenicity, while there was no significant correlation between the IEDB immunogenicity score and the proportion of T cells recognising a given epitope. Eight peptide-K^d^ epitopes were selected for inclusion in a tetramer panel to detect directly alloreactive CD8^+^ T cells. This panel bound over 30% of activated alloreactive CD8^+^ T cells after a prime-boost against K^d^. Moreover, the panel identified alloreactive CD8^+^ T cells within the graft infiltrate, spleen and draining lymph node during rejection of a K^d^-bearing heart graft. In conclusion, small animal studies have demonstrated the feasibility of high-throughput approaches for the discovery of pMHC epitopes recognised by directly alloreactive T cells. Translating this approach to the human setting is achievable and will yield both critical insights into the fundamental basis of alloreactivity and powerful tools for immune monitoring in transplantation.

## Introduction

Alloreactive T cells can recognise their cognate antigens via several different pathways (1). In the direct pathway of allorecognition, intact donor Major Histocompatibility Complex (MHC) molecules are recognised on the surface of donor cells. Conversely, donor MHC can be internalised and processed within recipient antigen-presenting cells (APC), resulting in the self-restricted presentation of peptides derived from the allogeneic donor MHC. This is referred to as the indirect pathway. A third pathway involves the transfer of intact donor MHC from donor cells to the surface of recipient APC and is termed semi-direct allorecognition.

Growing evidence indicates that the majority of directly alloreactive CD8^+^ T cells recognise epitopes comprising allogeneic donor MHC class I (MHC I) molecules complexed with one or more endogenous peptides (2–4). Distinguishing the highly immunogenic peptides which are recognised by a large proportion of recipient alloreactive cells from the remainder of the immunopeptidome is a daunting task. Until recently, most peptides recognised by directly alloreactive CD8^+^ T cells were discovered serendipitously, through their binding to individual T cell clones (5–8), but this does not always correlate with the extent of recognition by polyclonal responder populations. Lately, we developed a systematic pipeline for peptide-MHC (pMHC) epitope discovery, combining mass spectrometry-based determination of the immunopeptidome of transplantable organs with tetramer staining of alloreactive populations expanded *in vivo* (2, 9).

Our initial epitope discovery efforts focused on the endogenous peptide repertoire presented by H-2K^b^ (K^b^). We determined characteristics that favoured peptide recognition at a population level; high peptide abundance in the tissue(s) of interest, and ubiquitous expression across a range of different tissue and cell types (2). Here, we have applied these principles to the discovery of immunogenic self-peptides presented by a different allomorph, H-2K^d^ (K^d^), demonstrating that a panel of pMHC multimers selected based on strong expression in each of liver, skin and spleen can readily detect directly alloreactive T cells in the graft infiltrate, draining lymph node (DLN) and spleen during rejection of a K^d^-bearing heart graft. Methodological advances now permit the extension of systematic epitope discovery to additional allomorphs, target tissues and species; these will be discussed further.

## Results

### Identification of immunogenic K^d^-peptide epitopes

**Figure 1:**
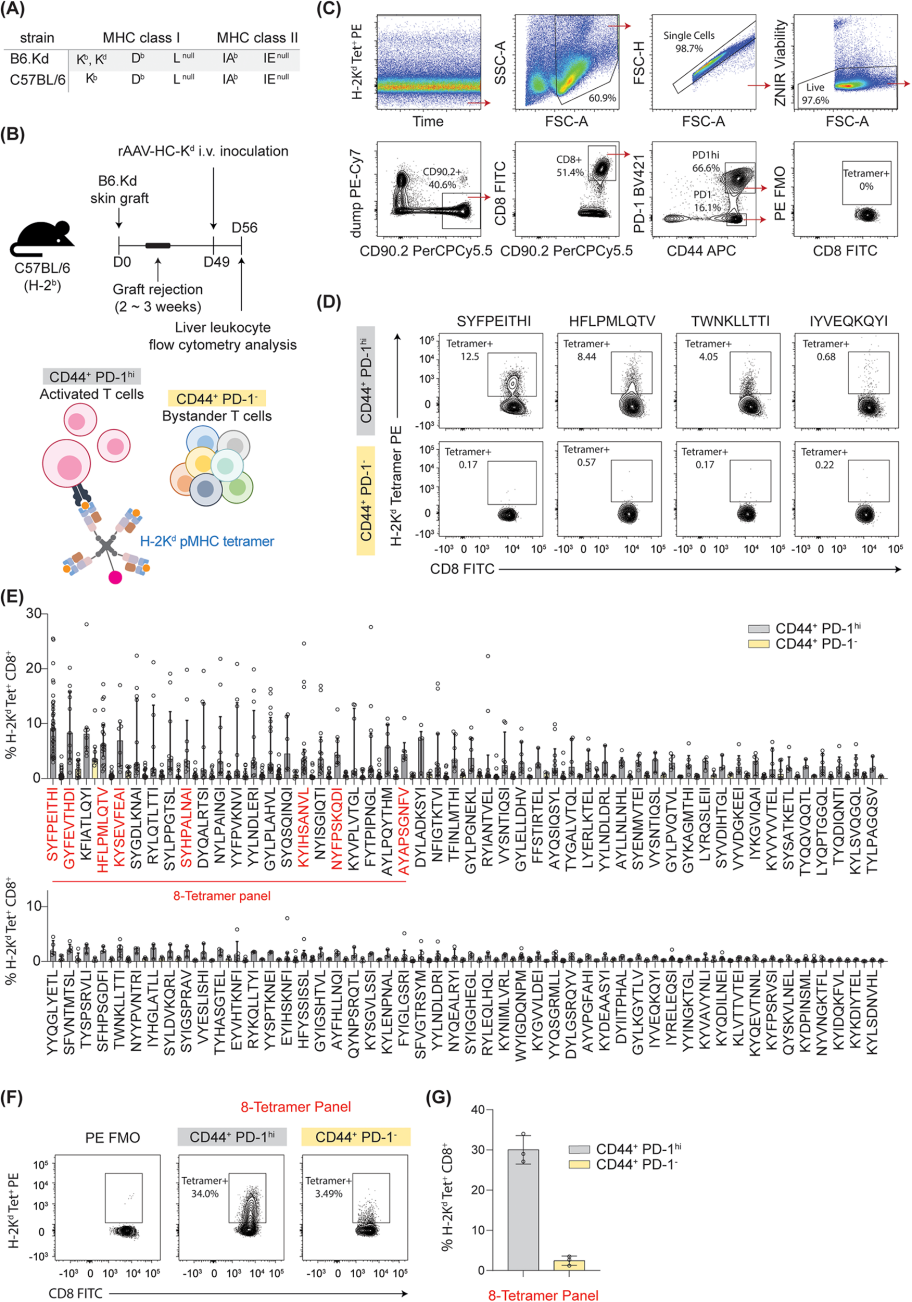
Identification of immunogenic peptide-K^d^ epitopes for directly alloreactive CD8^+^ T cells. Haplotypes of the donor and recipient strain are given at **(A)**. **(B)** Schematic illustrating the timecourse for *in vivo* priming and boosting of the response against K^d^. **(C)** Gating strategy. **(D)** Representative examples of CD8^+^ T cell recognition of different peptide-K^d^ epitopes. **(E)** Summary of T cell recognition of 100 K^d^-peptide epitopes. (**F-G)**. Binding of 8 tetramer panel to activated (PD-1^hi^) and bystander (PD-1^−^) cells after priming and boosting as described above. *n* = 3.

A set of 880 K^d^-restricted peptides had been detected across rejecting B6.K^d^ skin grafts, B6.K^d^ spleen cells and C57BL/6 hepatocytes transduced with AAV-K^d^ (2). From this set, 100 peptides were chosen for further evaluation, based on abundance (as estimated by spectral intensity) and predicted IC50 ≤ 500 nM [Net MHC pan 4.1 (10), Table 1]. MHC haplotypes of donor and recipient strains are shown in Figure 1A. Liver leukocyte populations enriched for activated alloreactive T cells were generated following priming of C57BL/6 mice with a B6.K^d^ skin graft, and boosting by inoculation with AAV-K^d^ (Figure 1B). On d7 after inoculation, liver leukocytes were isolated and stained using each of 100 individual K^d^-peptide tetramers to determine the proportion of CD8^+^ T cells recognising each epitope. (Figure 1B). The gating strategy is shown in Figure 1C. Alloreactive CD8^+^ T cells are CD44^+^ and PD-1^hi^, whereas bystander cells are PD-1^−^. Tetramer binding results are presented in Table 1 and Figure 1D,E. Of the 100 peptides screened, 22 were recognised by ≥5.0% of activated alloreactive T cells, with a ratio of ≥5 between binding of activated and bystander cells, and these were designated as highly immunogenic.

**Table 1:** H-2Kd-bound peptides screened for recognition by activated alloreactive CD8^+^ T cells.

Amino acid sequence	Log product spectral intensity	Mean PD-1hi (%)	Median PD-1hi (%)	Range PD-1hi (%)	Mean PD-1-(%)	Median PD-1-(%)	Range PD-1-(%)	Ratio PD-1hi vs. PD-1-
**SYFPEITHI**	27.94	10.11	9.12	(2.93–25.5)	0.73	0.44	(0–4.01)	13.85
**GYFEVTHDI**	23.27	9.73	8.32	(1.07–20.2)	1.79	1.7	(0.072–4.44)	5.44
KFIATLQYI	23.11	9.63	8.11	(4.12–28.1)	4.81	3.41	(2.36–12.4)	2
**HFLPMLQTV**	25.42	7.31	6.06	(1.3–17.2)	0.52	0.44	(0–2.12)	14.06
**KYSEVFEAI**	22.3	7.05	6.9	(1.16–17)	1.27	1.16	(0.23–2.22)	5.55
SYGDLKNAI	24.52	6.82	2.64	(0.5–22.4)	1	0.53	(0.12–2.86)	6.82
RYLQTLTTI	24.35	6.66	1.77	(0.72–21.2)	0.8	0.63	(0.075–2.52)	8.33
SYLPPGTSL	23.04	6.43	3.52	(0.93–19.1)	0.79	0.25	(0.13–2.54)	8.14
**SYHPALNAI**	23.81	6.41	3.22	(1.27–19.8)	0.84	0.6	(0.061–2.94)	7.63
DYQALRTSI	24.11	6.35	1.53	(0.15–19.6)	0.8	0.34	(0.058–2.4)	7.94
NYLPAINGI	23.01	6.34	3.07	(0.85–21.8)	0.74	0.31	(0.048–2.45)	8.57
YYFPVKNVI	23.96	6.24	1.33	(0.33–20.9)	0.85	0.48	(0.043–2.54)	7.34
YYLNDLERI	24.4	6.24	2.94	(0.5–19.9)	0.78	0.61	(0.03–2.42)	8
GYLPLAHVL	24.72	6.19	2.62	(1.19–16.3)	0.98	0.76	(0.078–4.38)	6.32
SYQSQINQI	23.01	5.87	4.52	(1.52–11.7)	0.72	0.65	(0–1.37)	8.15
**KYIHSANVL**	24.53	5.67	3.3	(0.92–24.6)	0.53	0.23	(0.057–2.79)	10.7
NYISGIQTI	23.37	5.46	3.56	(0.8–16.9)	0.6	0.25	(0.1–1.94)	9.1
**NYFPSKQDI**	23.76	5.45	4.27	(2.44–12.6)	0.65	0.51	(0.073–1.25)	8.38
KYVPLVTGL	23.09	5.35	1.37	(0.54–13.5)	0.7	0.3	(0.029–1.69)	7.64
FYTPIPNGL	23.36	5.33	1.59	(0.39–27.6)	0.85	0.47	(0.088–3.43)	6.27
AYLPQYTHM	23.65	5.32	5.77	(0.67–10.9)	0.65	0.48	(0.11–1.81)	8.18
**AYAPSGNFV**	22.38	5.28	4.38	(3.33–11)	0.56	0.54	(0.05–0.097)	9.43
DYLADKSYI	23.11	5.11	7.23	(0.29–9.75)	0.69	0.81	(0.08–1.39)	7.41
NFIGTKTVI	22.95	4.75	1.93	(0.19–17.3)	0.63	0.22	(0.026–2.38)	7.54
TFINLMTHI	23.29	4.58	3.27	(1.32–8.56)	0.73	0.66	(0.28–1.45)	6.27
GYLPGNEKL	23.41	4.42	3.68	(1.16–9.29)	0.71	0.62	(0.28–1.48)	6.23
RYIANTVEL	23.27	4.01	0.61	(0.38–22.3)	0.79	0.31	(0.086–4.18)	5.08
VYSNTIQSI	22.23	3.98	2.35	(0.85–10.4)	0.47	0.4	(0.091–1)	8.47
GYLELLDHV	23.85	3.84	2.88	(0.87–9.08)	0.48	0.31	(0.14–1.64)	8
FFSTIRTEL	23.84	3.45	2.69	(2.07–5.58)	0.76	0.94	(0.17–1.16)	4.54
AYQSIQSYL	23.64	3.42	1.9	(0.85–9.89)	0.95	0.21	(0.062–4.7)	3.6
TYGALVTQL	22.8	3.41	2.1	(0.73–7.45)	0.39	0.43	(0.076–0.64)	8.74
LYERLKTEL	23.8	3.31	2.73	(1.4–7.28)	0.72	0.43	(0.1–1.85)	4.6
YYLNDLDRL	22.74	3.22	2.96	(0.67–6.02)	0.32	0.34	(0.16–0.46)	10.06
AYLLNLNHL	23.09	3.16	2.99	(2.14–5.16)	0.45	0.33	(0.041–1.17)	7.02
SYENMVTEI	23.04	3.09	2.93	(1.48–4.52)	0.63	0.59	(0.23–1.03)	4.9
VYSNTIQSL	22.23	3.08	3.44	(1.08–4.72)	0.59	0.63	(0.28–0.85)	5.22
GYLPVQTVL	22.52	2.97	2.84	(1.53–4.59)	0.32	0.34	(0.22–0.38)	9.28
GYKAGMTHI	22.73	2.89	2.33	(0.68–6.41)	0.4	0.44	(0.66–0.095)	7.23
LYRQSLEII	23.64	2.87	3.12	(1.38–3.85)	0.75	0.57	(0.24–1.62)	3.83
SYVDIHTGL	23.45	2.69	2.11	(1.68–4.29)	0.72	0.74	(0.49–0.92)	3.74
VYVDGKEEI	22.53	2.66	2.08	(1.02–6.27)	0.56	0.57	(0.12–1.07)	4.75
IYKGVIQAI	22.32	2.64	3.15	(0.24–4.63)	0.4	0.33	(0.1–0.78)	6.6
KYVYVVTEL	23.29	2.63	1.61	(0.31–6.66)	1.42	0.79	(0.061–3.43)	1.85
SYSATKETL	22.77	2.56	1.86	(1.39–5.73)	0.44	0.42	(0.054–0.92)	5.82
TYQQVQQTL	22.49	2.54	2.08	(2.01–3.54)	0.43	0.41	(0.39–0.5)	5.91
LYQPTGGQL	22.59	2.54	2.15	(0.72–5.1)	0.41	0.35	(0.14–0.97)	6.2
TYQDIQNTI	23.15	2.52	2.11	(0.57–5.91)	0.34	0.26	(0.053–0.88)	7.41
KYLSVQGQL	22.47	2.47	1.69	(0.7–6.27)	0.56	0.49	(0.21–0.94)	4.41
TYLPAGQSV	22.52	2.46	1.91	(1.4–4.06)	0.36	0.29	(0.27–0.53)	6.83
YYQGLYETL	22.72	2.4	1.82	(1.19–4.49)	0.58	0.31	(0.2–1.09)	4.14
SFVNTMTSL	22.21	2.38	2.15	(1.65–3.71)	0.73	0.33	(0–2.34)	3.26
TYSPSRVLI	22.46	2.36	2.51	(1.47–3.1)	0.31	0.13	(0.12–0.69)	7.61
SFHPSGDFI	22.53	2.12	1.86	(1.73–2.76)	0.41	0.37	(0.19–0.67)	5.17
TWNKLLTTI	22.17	2.12	2.25	(0.52–4.06)	0.35	0.33	(0.039–0.71)	6.06
NYYPVNTRI	22.24	2.09	2.36	(1.48–2.42)	0.51	0.46	(0.33–0.74)	4.1
IYHGLATLL	22.26	2.01	2.45	(0.45–3.12)	0.55	0.56	(0.26–0.83)	3.65
SYLDVKQRL	22.41	1.88	1.8	(0.71–3.12)	0.48	0.45	(0.23–0.77)	3.92
SYIGSPRAV	22.76	1.87	2	(0.8–2.8)	0.22	0.16	(0.16–0.34)	8.5
VYESLISHI	22.5	1.84	1.62	(0.6–3.29)	0.21	0.22	(0.14–0.27)	8.76
TYHASGTEL	22.27	1.76	1.97	(1.05–2.26)	0.52	0.38	(0.29–0.89)	3.38
EYVHTKNFI	23.13	1.76	1.05	(0.18–5.83)	0.3	0.15	(0.04–0.87)	5.87
RYKQLLTYI	22.9	1.71	1.71	(1.64–1.78)	0.35	0.32	(0.28–0.44)	4.89
YYSPTKNEI	22.93	1.65	1.72	(1.47–1.77)	0.31	0.26	(0.24–0.42)	5.32
EYIHSKNFI	24.52	1.64	0.55	(0.22–7.89)	0.5	0.25	(0.034–1.92)	3.28
HFYSSISSL	23.11	1.58	1.24	(0.77–4.08)	0.43	0.29	(0.13–0.83)	3.67
GYIGSHTVL	22.88	1.56	1.42	(0.89–2.38)	0.44	0.46	(0.37–0.49)	3.55
AYFHLLNQI	22.85	1.5	1.53	(1.09–1.89)	0.37	0.32	(0.19–0.61)	4.05
QYNPSRQTL	22.82	1.5	1.15	(0.71–2.63)	0.23	0.06	(0.034–0.61)	6.52
KYSGVLSSI	22.86	1.38	1.46	(1.19–1.48)	0.31	0.35	(0.22–0.37)	4.45
KYLENPNAL	22.5	1.36	1.07	(0.83–2.18)	0.35	0.25	(0.22–0.59)	3.89
FYIGLGSRI	23.09	1.34	0.66	(0.23–5.14)	0.17	0.11	(0–0.58)	7.88
SFVGTRSYM	22.43	1.28	0.83	(0.3–3.7)	0.17	0.15	(0.04–0.34)	7.53
YYLNDLDRI	22.74	1.21	0.9	(0.21–3.63)	0.27	0.26	(0.13–0.43)	4.48
NYQEALRYI	22.24	1.2	0.78	(0.51–2.72)	0.27	0.27	(0.082–0.46)	4.44
SYIGGHEGL	22.2	1.15	1.21	(0.71–1.53)	0.24	0.12	(0.074–0.52)	4.79
RYLEQLHQL	23.37	1.14	0.9	(0.87–1.65)	0.28	0.21	(0.18–0.46)	4.07
KYNIMLVRL	22.19	1.13	1.25	(0.83–1.3)	0.4	0.34	(0.22–0.64)	2.83
WYIGDQNPM	25.2	1.08	0.86	(0.49–2.41)	0.29	0.28	(0.091–0.62)	3.72
KYGVVLDEI	25.56	1.02	0.98	(0.48–1.6)	0.13	0.13	(0.11–0.15)	7.85
YYQSGRMLL	22.97	0.93	0.88	(0.23–1.79)	0.15	0.12	(0–0.31)	6.2
DYLGSRQYV	23.24	0.9	0.91	(0.42–1.34)	0.15	0.14	(0.029–0.31)	6
AYVPGFAHI	25.31	0.89	0.97	(0.54–1.17)	0.19	0.19	(0.19–0.19)	4.68
KYDEAASYI	22.34	0.83	0.57	(0.56–1.35)	0.36	0.39	(0.16–0.54)	2.31
DYIITPHAL	22.57	0.82	0.99	(0.47–0.99)	0.24	0.24	(0.2–0.29)	3.42
GYLKGYTLV	22.41	0.81	0.69	(0.62–1.13)	0.21	0.17	(0.077–0.38)	3.9
IYVEQKQYI	22.23	0.79	0.73	(0.71–0.94)	0.22	0.22	(0.18–0.25)	3.59
IYRELEQSI	22.7	0.77	0.52	(0.42–2.16)	0.2	0.2	(0.072–0.35)	3.85
YYINGKTGL	22.65	0.73	0.71	(0.59–0.9)	0.26	0.25	(0.23–0.3)	2.81
KYVAVYNLI	22.82	0.68	0.43	(0.27–1.35)	0.37	0.18	(0.097–0.83)	1.84
KYQDILNEI	25.43	0.68	0.51	(0.33–1.66)	0.17	0.15	(0.09–0.3)	4
KLVTTVTEI	22.26	0.58	0.75	(0.19–0.79)	0.41	0.47	(0.17–0.58)	1.41
KYQEVTNNL	24.83	0.58	0.49	(0.26–0.98)	0.18	0.22	(0.073–0.24)	3.22
KYFPSRVSI	23.89	0.56	0.52	(0.32–0.84)	0.17	0.17	(0.12–0.23)	3.29
QYSKVLNEL	22.8	0.54	0.4	(0.24–0.99)	0.1	0.11	(0–0.2)	5.4
KYDPINSML	22.26	0.49	0.36	(0.27–0.99)	0.3	0.12	(0.03–1.05)	1.63
NYVNGKTFL	24.08	0.49	0.42	(0.17–0.87)	0.18	0.17	(0.13–0.24)	2.72
KYIDQKFVL	22.36	0.32	0.23	(0.22–0.5)	0.21	0.15	(0.094–0.38)	1.52
KYKDIYTEL	24.85	0.3	0.34	(0.17–0.4)	0.06	0.03	(0.026–0.12)	5
KYLSDNVHL	22.41	0.26	0.21	(0.18–0.38)	0.15	0.15	(0.023–0.27)	1.73

The peptide sequence and log product of the spectral intensity across spleen, grafted tail skin and transduced hepatocytes are shown, alongside the proportion of cells bound by each Kd-peptide tetramer from activated (PD-1hi) and bystander (PD-1^−^) populations of CD8^+^ T cells. Peptides included in the staining panel are shown in boldface. The mass spectrometry data for these peptides have been deposited to the ProteomeXchange Consortium via the PRIDE partner repository with accession number PXD022695.

A further 34 peptides were recognised by between 2.0 and 4.99% of activated CD8^+^ cells (moderately immunogenic), while 43 peptides were recognised by ≤1.99% of activated CD8^+^ cells (non-immunogenic). These proportions imply significant cross-reactivity in recognition of different pMHC species by alloreactive T cells. Representative examples of tetramer staining for each of these groups are shown in Figure 1D, while the summary of tetramer binding results appears in Figure 1E. One peptide, KFIATLQYI, was consistently recognised by both activated (9.63%) and bystander cells (4.81%). Eight peptides were selected for inclusion into a panel for detection of alloreactive CD8^+^ T cells (Figure 1F,G). This panel detected >30% of activated CD8^+^ T cells expanded using the same prime-boost strategy outlined above.

### Factors contributing to peptide immunogenicity

**Figure 2:**
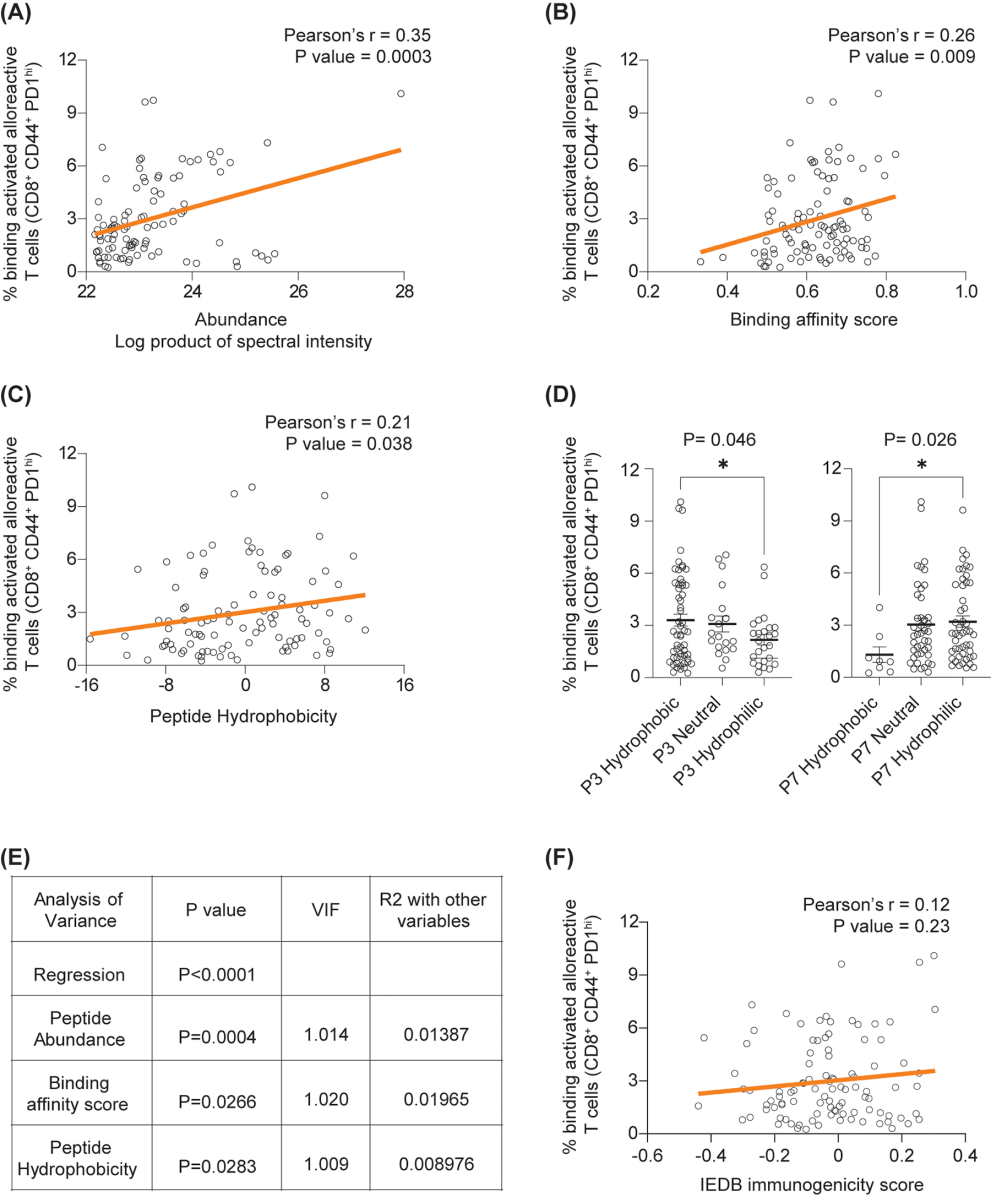
Factors contributing to peptide immunogenicity. Abundance **(A)**, predicted binding affinity **(B)** and overall hydrophobicity **(C)** were each significantly correlated with recognition by alloreactive CD8^+^ T cells. **(D)** Hydrophobicity at P3 was positively correlated with TCR binding while the converse was true at P7. These relationships were preserved following multivariable analysis **(E)**. **(F)** No significant correlation was demonstrated between the IEDB immunogenicity score and extent of binding to alloreactive CD8^+^ populations.

Similar to our earlier findings for K^b^-bound peptides (2), the strongest predictor of immunogenicity for the peptide cargo of K^d^ was peptide abundance, here estimated as the product of the peptide spectral intensity across rejecting skin grafts, transduced hepatocytes and spleen (Figure 2A). Both abundance and T cell binding values for K^d^-SYFPEITHI were considerably higher than for the remaining peptides. Robustness of the correlation was tested by a) converting numerical values to ranks and using a non-parametric test (Spearman's correlation; Supplementary Figure S1A) and b) removing Kd-SYFPEITHI. In both cases, significant correlations were maintained. The binding affinity (BA) score from NetMHC pan 4.1 (10) also correlated with immunogenicity (Figure 2B), as did the overall peptide hydrophobicity as measured by the Kyte-Doolittle scale (Figure 2C) (11). As for abundance, these correlations remained significant when Spearman's test was employed. Hydrophobicity at P3 or P6 was associated with increased immunogenicity, while the opposite was true of hydrophobicity at P7 (Figure 2D, Supplementary Figure S1B). Hydrophobicity at other non-anchor positions was not significantly associated with immunogenicity (Supplementary Figure S1B). Multivariable analysis showed that peptide abundance, BA score and overall hydrophobicity were independent of each other and all three were significantly associated with peptide recognition by activated alloreactive CD8^+^ T cells (Figure 2E).

In contrast to these findings, there was no significant correlation between the IEDB immunogenicity score (12) and the proportion of CD8^+^ T cells recognising a given peptide presented by H-2K^d^ (Figure 2F). H-2K^b^-presented peptides are shorter than those favoured by H-2K^d^, and biochemically distinct, with an unusual binding motif featuring an aromatic residue at peptide position (P)5. In this instance, neither the overall hydrophobicity (Supplementary Figure S1C) nor the hydrophobicity score for any individual residue, was correlated with immunogenicity. The IEDB immunogenicity score, designed for 9-mers, was not applied to the H-2K^b^ 8-mer peptide dataset.

### A core K^d^-peptide tetramer panel identifies alloreactive T cells during heart graft rejection

**Figure 3:**
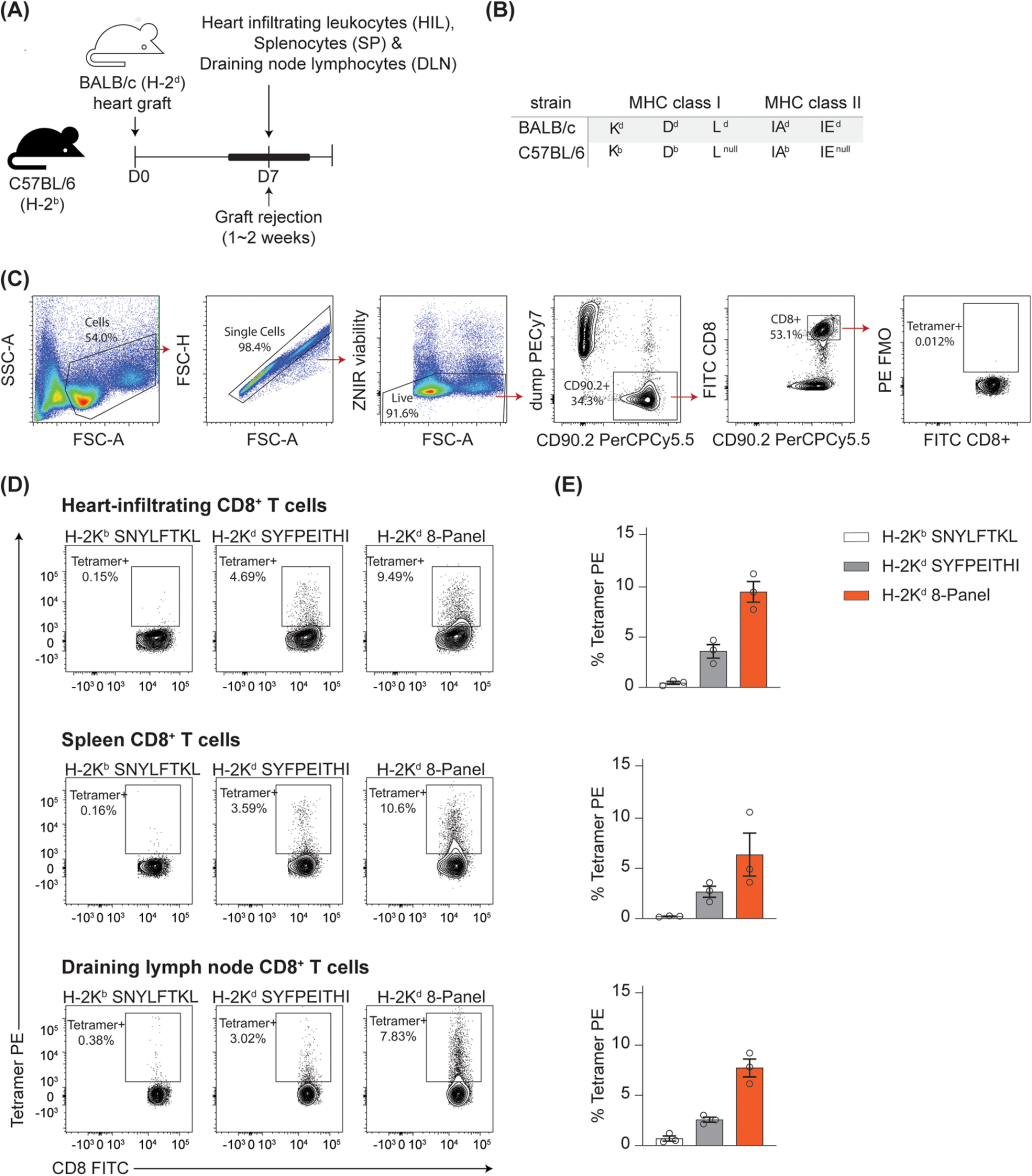
A core panel of peptide-K^d^ tetramers identifies alloreactive CD8^+^ T cells in a fully allogeneic model of heart transplant rejection. **(A)** Cartoon depicting the experimental model. **(B)** Haplotypes of donor and recipient strain. **(C)** Gating Strategy. **(D)** Representative flow plots showing tetramer binding to Heart-infiltrating CD8^+^ T cells, splenocytes and draining node lymphocytes. Binding to the 8-tetramer panel is shown alongside binding of the dominant epitope K^d^-SYFPEITHI, and the negative control pMHC K^b^-SNYLFTKL **(E)**. Summary of data presented at **(D)**, *n* = 3.

To determine whether our core 8-tetramer panel would be able to detect alloreactive T cells responding to a different target tissue in the setting of a full allogeneic mismatch, BALB/c hearts were transplanted into C57BL/6 recipients (Figure 3A). The haplotypes of this donor-recipient strain combination are shown in Figure 3B, while Figure 3C depicts the gating strategy. Heart-infiltrating leukocytes, splenocytes and lymphocytes from the parathymic (draining) nodes (DLN) were isolated on day 7 post-transplantation and stained with the 8-tetramer panel described above (Figure 3D). 9.51 ± 1.03, 6.38 ± 2.14 and 7.73 ± 0.89% of CD8^+^ T cells from rejecting heart, spleen and DLN respectively bound the 8-tetramer panel, compared with 3.59 ± 0.68, 2.68 ± 0.56 and 2.58 ± 0.25% binding a single tetramer of the dominant epitope K^d^-SYFPEITHI. Binding to the control syngeneic tetramer of K^b^ loaded with the abundant self-peptide SNYLFTKL was less than 0.75% for CD8^+^ T cells isolated from each tissue (Figure 3E).

Tetramer-positive proportions were consistently higher in activated T cells than in non-activated cells. In the graft infiltrate, (Supplementary Figure S2) 93.1% of CD8^+^ T cells were activated (CD44 ^+^ PD-1^hi^); of these, 10.07 ± 1.1% bound the tetramer panel, compared with 1.25 ± 0.14% of cells that were PD-1^−^. Conversely, in the DLN, the majority of cells were naïve (64.1% CD44^−^), with 6.45% CD44^+^ PD-1^hi^ (Supplementary Figure S3). The proportion of tetramer-positive activated cells was very high at 31.7 ± 4.08% with 4.14 ± 0.82% of naïve cells binding the tetramer panel (Supplementary Figure S3). Roughly equal numbers of spleen CD8^+^ T cells were found in the activated and naïve compartments (Supplementary Figure S4). In the spleen, tetramer-positive fractions were 12.99% ± 3.06 and 3.16 ± 1.74% of activated and naïve cells respectively.

## Discussion

Determining the pMHC specificity of directly alloreactive T cells has been a longstanding, intractable problem in transplantation. Systematic discovery of these epitopes will enable deeper understanding of the interactions between alloreactive T cells and their ligands and provide tools to improve risk stratification and immune monitoring post-transplantation. Here, we show that the approach employed to determine immunogenic K^b^-peptide epitopes for recipients from H-2^k^ or H-2^d^ genetic backgrounds can be extended to the additional combination of the donor allomorph K^d^ with the H-2^b^ recipient haplotype, and that the core panel comprising abundant peptides shared between liver, skin and spleen allows detection of alloreactive T cells responding to a fully allogeneic transplanted heart where the immunopeptidome is expected to include a set of organ-specific peptides presented by K^d^ in addition to the shared peptides, and further H-2L^d^ (L^d^) or H-2D^d^ (D^d^)-restricted epitopes.

T cells responding to the dominant epitope K^d^-SYFPEITHI alone accounted for one third to one half of all cells detected by the 8-tetramer panel. The overall proportion detected by the panel was roughly half of that which would be expected on the basis of a simple summation of all the tetramer-positive frequencies, consistent with cross-reactivity between different epitopes. Expanding the core panel with tissue-specific epitopes and inclusion of additional donor MHC types would likely augment the proportion of cells detected. QL9 (QLSPFPFDL) is an L^d^-restricted peptide derived from the source protein ɑ-ketoglutarate dehydrogenase that is recognised by the alloreactive 2C TCR (8). Cohen et al. recently demonstrated that L^d^-QL9 tetramers could be used to track C57BL/6 CD8^+^ T cells responding to BALB/c skin grafts (3), and this specificity would be an obvious choice for addition to the core K^d^-peptide panel. Systematic identification of further L^d^ and D^d^-restricted epitopes requires surveying the immunopeptidome of a range of transplantable organs followed by candidate peptide selection and screening.

Predictions of peptide immunogenicity based on features including abundance, physicochemical and structural characteristics can guide the selection of peptides for empirical screening, reducing the size of the “haystack” in which the “needles” must be sought. Peptide abundance has been correlated with immunogenicity for viral epitopes (13) as well as the self-peptides evaluated here and in our preceding study (2). Conversely, biochemical characteristics that predict recognition in certain contexts may not be universally applicable. The IEDB immunogenicity predictor was trained primarily on pathogen-derived epitopes (12). Using this predictor, viral peptides consistently received higher immunogenicity scores than self-peptides (12) and thus this tool may not be generalisable to prediction of immunogenicity for self-peptides presented by allogeneic MHC I molecules.

Novel methods for generating libraries of pMHC monomers permit the extension of this approach beyond the range of MHC Class I types for which peptide exchange or loading methodologies are available (14). Moreover, peptide exchange for MHC class II molecules has recently been demonstrated using the combination of recombinant class II with covalently-linked cleavable peptides and soluble HLA-DM (15), facilitating the additional detection of directly-alloreactive CD4^+^ T cells.

Methodology developed for murine studies can be adapted for discovery of peptide-HLA (Human Leukocyte Antigen) epitopes recognised by human alloreactive T cells. In place of *in vivo* expansion, responder cells are co-cultured in a mixed lymphocyte reaction, or with artificial antigen-presenting cells expressing the allomorph of interest (4, 16–18). Zhang et al. have recently demonstrated that externally pulsing artificial antigen presenting cells with a pool of abundant peptides expressed by the target tissue of interest (in this case renal tubular cells) was effective in expanding alloreactive responder cells prior to screening these peptides for immunogenicity, resulting in the identification of two peptides recognised by CD8^+^ T cells from multiple individuals (4). These approaches rely on knowledge of the tissue immunopeptidome under relevant conditions. To this end, detailed profiling of the immunopeptidome of transplantable organs procured from organ donors, and including not only constitutively-expressed conventional linear peptides but immunoproteasome-dependent, cryptic, spliced and post-translationally modified peptides will be important (19).

In conclusion, small animal studies have demonstrated the feasibility of high-throughput approaches for the discovery of pMHC epitopes recognised by directly alloreactive T cells. Translating this approach to the human setting is achievable and will yield both critical insights into the fundamental basis of alloreactivity and powerful tools for immune monitoring in transplantation.

## Methods

### Peptides, antibodies and reagents

Peptides used in this study were synthesised with an average of 98% purity (GL Biochem Shanghai Ltd.). 10% DMSO was used in reconstituting lyophilised peptides and aliquots were stored at −80°C. 5 mM stock of Dasatinib (Sigma-Aldrich, catalogue# CDS023389) reconstituted in DMSO was stored at −80°C. Primary and secondary antibodies used in this study are summarised in Supplementary Table S1.

### Mice

C57BL/6J^Arc^ (H-2^b^) and BALB/c^Arc^ (H-2^d^) mice (herein termed C57BL/6 and BALB/c) were purchased from the Animal Resources Centre, Perth, Australia. B6.Kd mice (20, 21) express an H-2K^d^ transgene ubiquitously on a C57BL/6 (H-2^b^) background, and were bred at Australian Bioresources, Moss Vale, Australia. B6.Kd mice were backcrossed for 4 generations to C57BL/6J^Arc^, prior to use. 8–12 week old male mice were used in this study.

### Skin transplantation

Skin transplantation was performed as outlined previously (21). C57BL/6 recipient mice received full-thickness grafts of 1 × 1 cm tail skin from B6.Kd donor mice. Grafts were deemed rejected when less than 20% of the viable skin graft remained.

### AAV vectors

AAV vectors encoding H-2K^d^ were prepared as previously described (21). Briefly, H-2K^d^ cDNA was cloned into the pAM2AA backbone incorporating the liver-specific human *α*-1 antitrypsin promoter and human ApoE enhancer flanked by AAV2 inverted terminal repeats. This construct was packaged into an AAV2/8 vector, purified, and quantitated by the Vector and Genome Engineering Facility, Children's Medical Research Institute, Westmead, Australia. Vector aliquots were stored at −80°C. AAV-K^d^ was used at a dose of 5 × 10^11^ vgc/mouse.

### Heterotopic heart transplantation

The heart transplant procedure was carried out according to a published protocol (22). Donor hearts were perfused with 1.0 ml of cold heparinised saline through the inferior vena cava (IVC). The donor heart was retrieved and then stored in cold saline on ice prior to transplantation. The donor aorta and pulmonary artery were anastomosed end to side to the recipient aorta and IVC, respectively. Following the release of cross-clamps, haemostasis was achieved using gentle pressure with a cotton bud. C57BL/6 mice (H-2^b^) received fully mismatched allogeneic heart grafts from BALB/c donor mice (H-2^d^).

### Leukocyte isolation from liver, heart, spleen, and draining lymph nodes

For liver leukocyte isolation, the IVC was cannulated and the hepatic portal vein was transected. The liver was flushed with 20 ml of PBS at RT and after gallbladder removal, the liver was mashed through a 100 μm cell strainer and washed through with cold RPMI-1640 medium supplemented with L-glutamine (Lonza, catalogue# 12-702F) and 2% FCS (Sigma-Aldrich, catalogue# 13K179) (RMPI/FCS2 medium). The liver slurry was centrifuged at 400 *g* for 10 min and washed twice, then purified using isotonic Percoll PLUS (GE Healthcare Life Sciences) gradient separation - centrifuged at 500 *g* for 15 min at RT. The liver leukocyte pellet was collected, washed twice, and resuspended in red cell lysis buffer for 2 min at RT. Cells were used following two further washes with PBS containing 2% FCS.

For isolation of heart infiltrating leukocytes, the heart grafts were flushed by injecting 3 ml of cold PBS slowly into the abdominal aorta. The grafts were resected and stored in cold DMEM (Lonza, catalogue# BW12-709F). Grafts were washed with cold PBS and then transected lengthways. The bisected heart grafts were washed again with cold PBS. 500 μl of DMEM containing 400 U/ml collagenase Type II (Gibco, catalogue# 17101015) and 48 U/ml DNase I (Thermo Scientific, catalogue# EN0525) was injected directly into the parenchyma of the bisected heart grafts at multiple sites. Hearts were incubated for 10 min at 37°C with gentle shaking.

Following this, the grafts were diced and incubated further with 4.5 ml of DMEM containing collagenase Type II and 48 U/ml DNase I for 1 h at 37°C with gentle shaking. Digested heart tissue was gently pushed through a 70 μm nylon mesh strainer. Dissociated cells were washed with DMEM medium containing 15% FCS (DMEM/FCS15). The cells were centrifuged at 300 *g* for 3 min and then resuspended in 42% isotonic Percoll PLUS (GE Healthcare Life Sciences)/PBS. The resuspended cells were centrifuged at 800 *g* for 20 min at RT. The supernatant was discarded, and the heart infiltrating leukocyte pellet was then resuspended in red cell lysis buffer for 2 min at RT. Cells were used following two further washes with PBS containing 2% FCS. This method was adapted from Prosser et al. (23).

To obtain splenocytes, the spleen was pressed through a 70 μm nylon mesh strainer, washed and resuspended in red cell lysis buffer for 2 min at RT. The splenocytes were washed twice before use. To isolate lymphocytes from draining lymph nodes, the nodes were ruptured through a 40 μm nylon mesh strainer and then prepared as for splenocytes, with the omission of the red cell lysis step.

### pMHC multimer preparation

BioLegend Flex-T™ H2-K^d^ Monomer UVX (BioLegend, custom order) kits were utilised to generate biotinylated K^d^ monomers incorporating the selected peptides. These monomers were then assembled with PE-streptavidin (BioLegend, catalogue# 405203). 20 μl of selected peptide at 400 μM concentration was added to 20 μl of Flex-T monomer. The mixture was illuminated with long-wave 366 nm UV light on ice for 30 min, then incubated at 37°C for 30 min in the dark. The final exchanged monomer solution is at 2 μM concentration.

To assemble PE-conjugated tetramers, 2.0 μl of PE-streptavidin at 0.2 mg/ml concentration (BioLegend, catalogue# 405203) was added to 18 μl of the exchanged monomer. The reaction mixture was incubated at 4°C for 30 min protected from light. Blocking solution was prepared by mixing 1.6 μl of 50 mM D-Biotin (Sigma-Aldrich, catalogue# 711610) and 6 μl of 10% (w/v) sodium azide (Sigma-Aldrich, catalogue# S2002-5G) in 192.4 μl of PBS. 1.4 μl of the blocking solution was added to the reaction mixture and incubated at 4°C for at least 16 h protected from light. 5.8 μl of the PE-conjugated tetramer (approximately 0.5 μg) was used for each single test.

### pMHC multimer staining

The pMHC multimer staining method was adapted from Dolton et al. (24). Cells were first incubated with a protein kinase inhibitor, 50 nM dasatinib, in staining buffer (2% FCS in PBS) for 30 min at 37°C. PE-conjugated tetramers were centrifuged at 16,000 *g* for 1 min to remove aggregates. Cells were stained with 0.5 μg of pMHC tetramer in 50 *μ*l for 30 min at 4°C. Following pMHC multimer staining, the cells were washed with cold staining buffer twice. Samples were incubated with mouse Fc Block (BD Biosciences, catalogue# 553141) for 10 min at 4°C and mouse anti-PE antibody was added at 0.5 μg/100 μl. The cells were washed and incubated with a cocktail of antibodies against surface markers for 30 min on ice (Supplementary Table S1). Cells were washed twice with PBS before staining with viability dyes Zombie NIR (BioLegend, catalogue# 423105) for 15 min at RT. Cells were then washed with staining buffer. Sample data was acquired using either of the Fortessa X-20 or LSR-II (both BD Biosciences) instruments and analysed using FlowJo v10.

### Statistical analysis and data visualisation

Data are represented as mean ± SEM unless otherwise stated. Unpaired Student's *t*-tests were performed to calculate statistical differences in a single variable between the means of two groups. The relationships between overall peptide abundance, binding affinity score or hydrophobicity score and alloreactive T cell binding were analysed using linear regression and Pearson correlation tests. Spearman's correlation was performed as an additional test for robustness. *P* < 0.05 was considered significant. Multivariable analysis using least squares regression was then carried out. Statistical tests were performed using GraphPad Prism version 8.01 (GraphPad Software, La Jolla CA).

### Study approvals

All animal procedures were approved by the University of Sydney Animal Ethics Committee (protocol 2017/1253 and protocol 2022/2092) and carried out in accordance with the Australian code for the care and use of animals for scientific purposes. The use of genetically-modified mice was covered by University of Sydney Institutional Biosafety Committee approvals 18NO13 and 23NO23, while AAV vector production and use was approved under NLRDs 17NO28 and 22NO12.

## Data Availability

The datasets presented in this study can be found in online repositories. The names of the repository/repositories and accession number(s) can be found in the article/Supplementary Material.
