# Supplementary material for: Discovery of conserved peptide-MHC epitopes for directly alloreactive CD8+ T cells
Source: Front Transplant. 2025 Jan 29;4:1525003. doi: 10.3389/frtra.2025.1525003 (PMC11814428; doi:10.3389/frtra.2025.1525003)
Supplement: Supplementary file 1 [file Datasheet1.pdf]

A

Parameter	Pearson's R	P value	Spearman's R	P value
Abundance	0.3532	0.0003	0.3022	0.0022
BA score	0.2596	0.0091	0.2345	0.0189
Overall hydrophobicity	0.2079	0.0379	0.2229	0.0258

B

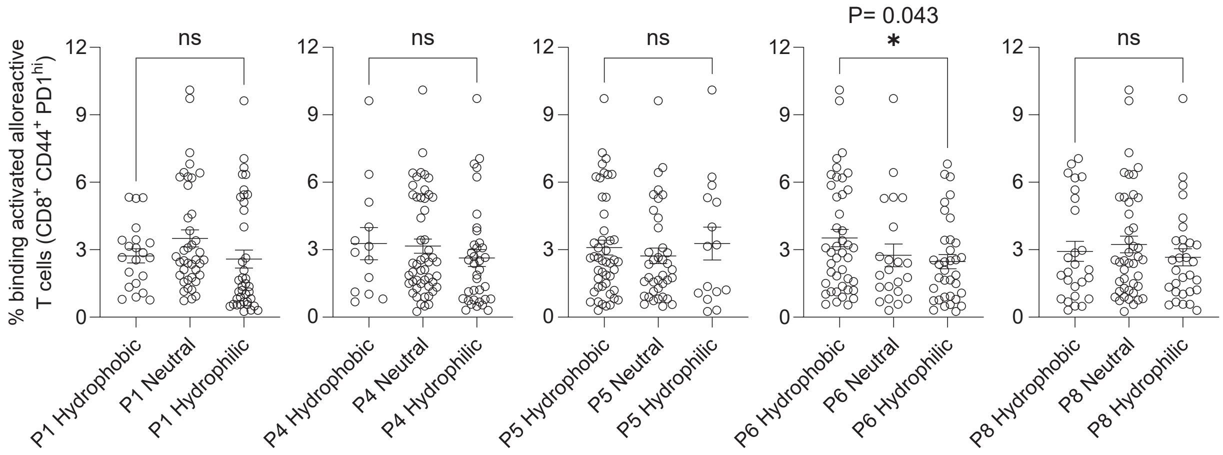

C

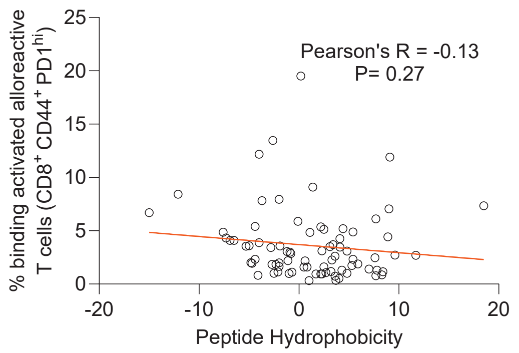

Figure S1. Results obtained for H-2K^{d}-bound peptides using Pearson's correlation were robust to the conversion of numerical values to ranks and non-parametric testing (Spearman's correlation)(**A**). Hydrophobicity at P6 but not other non-anchor positions was significantly associated with immunogenicity (**B**). Overall hydrophobicity did not predict immunogenicity for H-2K^{b}-presented peptides (**C**).

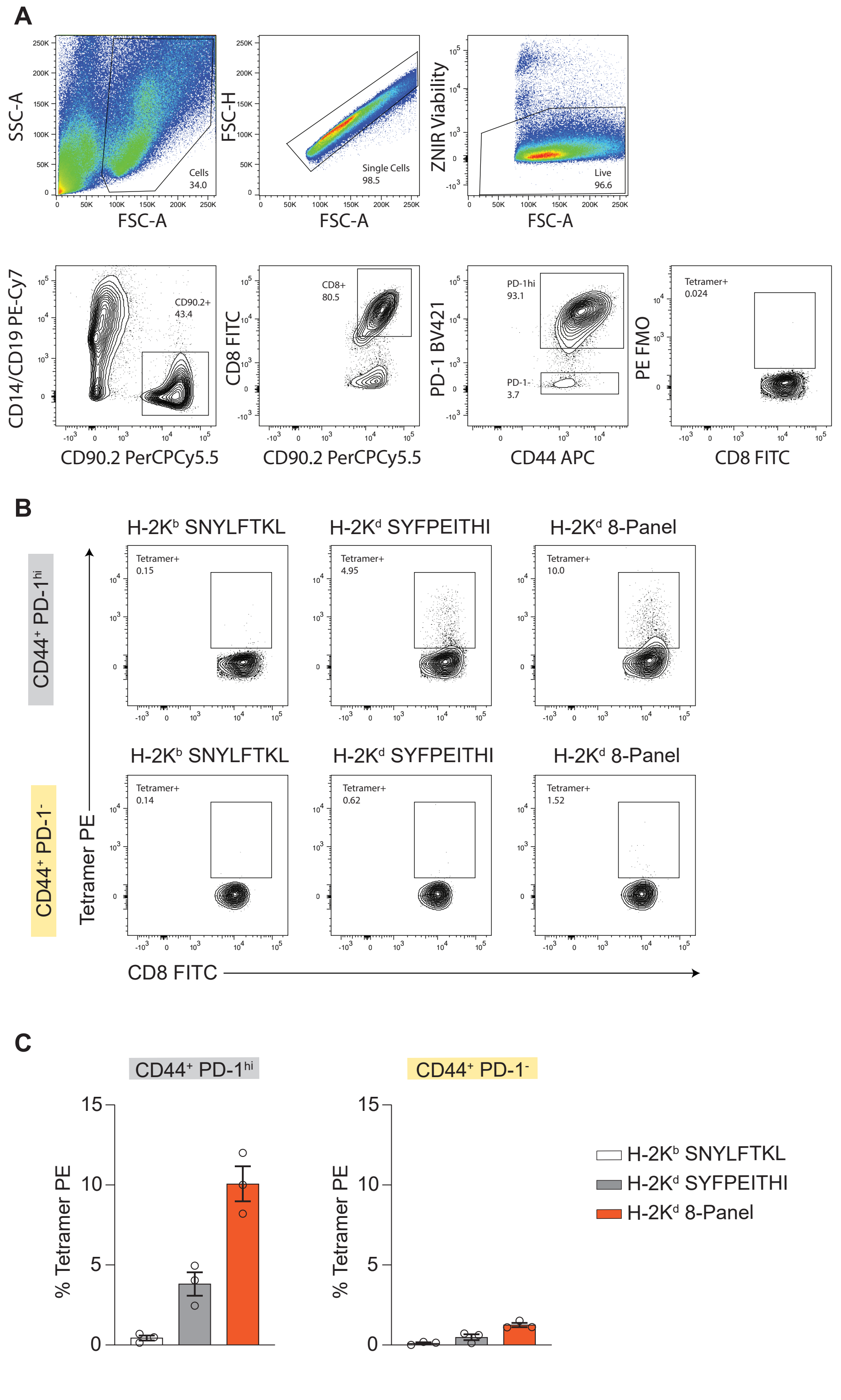

Figure S2. Tetramer binding to subpopulations of heart-infiltrating CD8^{+} T cells.

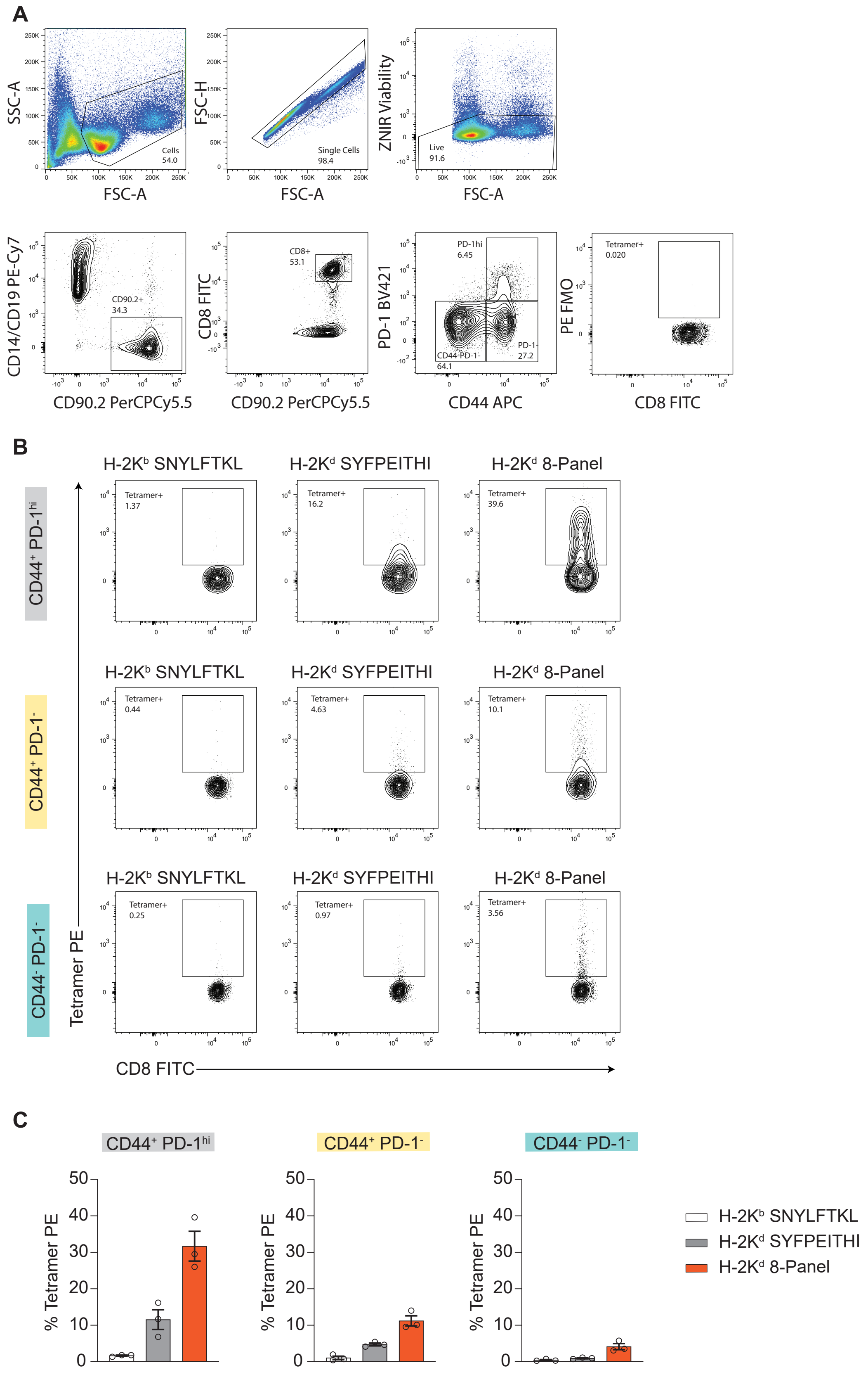

Figure S3. Tetramer binding to subpopulations of CD8^{+} T cells from the draining Lymph node (DLN).

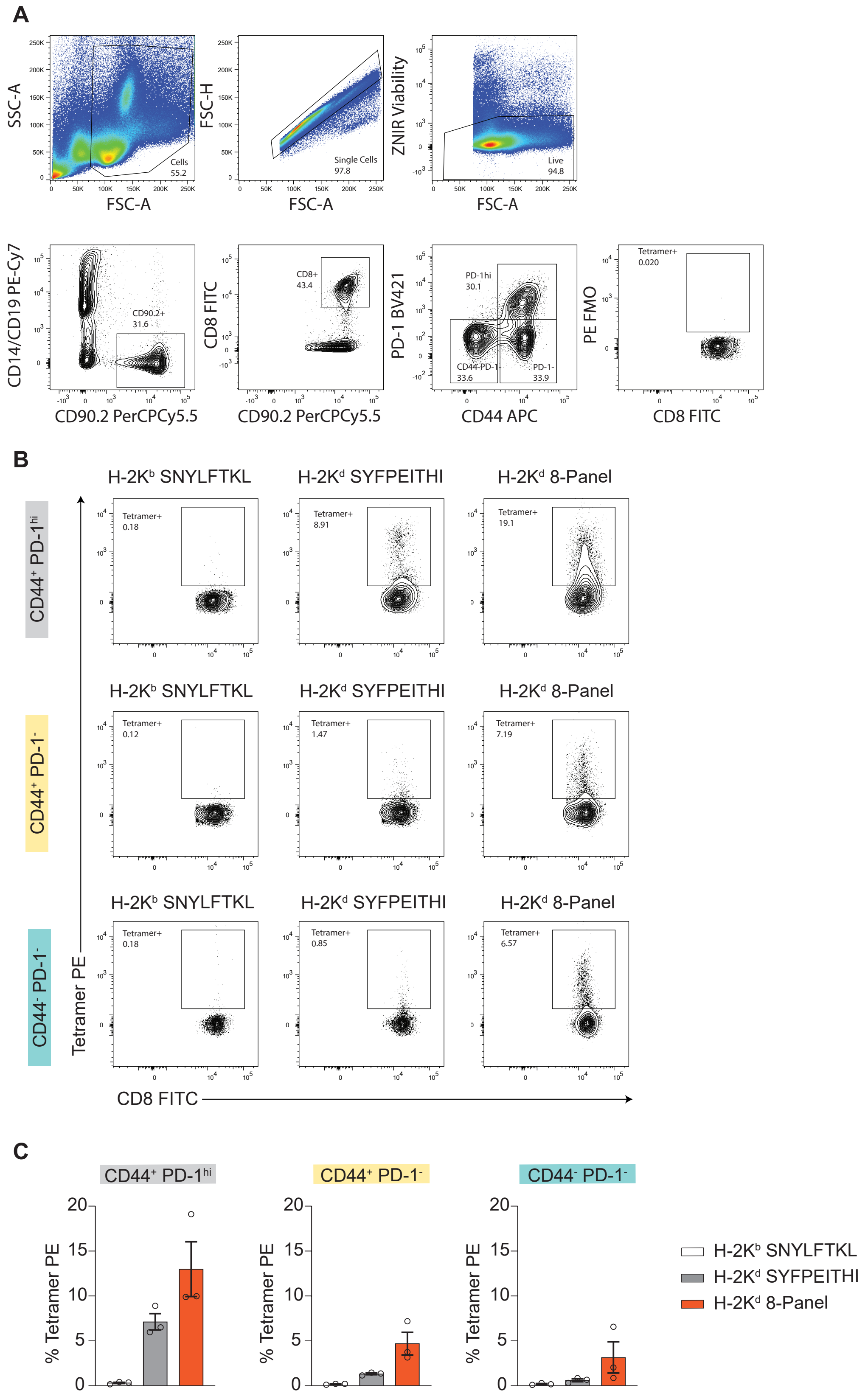

Figure S4. Tetramer binding to subpopulations of $CD8^+$ T cells from spleen.

Antibodies used for multimer staining				
Antibody Target	Clone	Format	Supplier	Catalogue #
CD8a	KT-15	FITC	Invitrogen	MA5-16760
CD90.2	53-2.1	PerCPCy5.5	BioLegend	140322
CD44	IM7	APC	BioLegend	103012
PD-1	29F.1A12	BV421	BioLegend	135218
CD19	6D5	PECy7	BioLegend	115520
CD14	Sa14-2	PECy7	BioLegend	740357
PE	PE001	purified	BioLegend	408102

Table S1: Summary of antibodies and reagents used in this study.
